# Noninvasive, near-field terahertz imaging of hidden objects using a single-pixel detector

**DOI:** 10.1126/sciadv.1600190

**Published:** 2016-06-03

**Authors:** Rayko Ivanov Stantchev, Baoqing Sun, Sam M. Hornett, Peter A. Hobson, Graham M. Gibson, Miles J. Padgett, Euan Hendry

**Affiliations:** 1 School of Physics, University of Exeter, Stocker Road, Exeter EX4 4QL, UK.; 2 School of Physics and Astronomy, University of Glasgow, Glasgow G12 8QQ, UK.; 3 QinetiQ Limited, Cody Technology Park, Ively Road, Farnborough GU14 0LX, UK.

**Keywords:** Compressed sensing, terahertz, near-field, noninvasive, single-pixel detection, optics, imaging

## Abstract

Terahertz (THz) imaging can see through otherwise opaque materials. However, because of the long wavelengths of THz radiation (λ = 400 μm at 0.75 THz), far-field THz imaging techniques suffer from low resolution compared to visible wavelengths. We demonstrate noninvasive, near-field THz imaging with subwavelength resolution. We project a time-varying, intense (>100 μJ/cm^2^) optical pattern onto a silicon wafer, which spatially modulates the transmission of synchronous pulse of THz radiation. An unknown object is placed on the hidden side of the silicon, and the far-field THz transmission corresponding to each mask is recorded by a single-element detector. Knowledge of the patterns and of the corresponding detector signal are combined to give an image of the object. Using this technique, we image a printed circuit board on the underside of a 115-μm-thick silicon wafer with ~100-μm (λ/4) resolution. With subwavelength resolution and the inherent sensitivity to local conductivity, it is possible to detect fissures in the circuitry wiring of a few micrometers in size. THz imaging systems of this type will have other uses too, where noninvasive measurement or imaging of concealed structures is necessary, such as in semiconductor manufacturing or in ex vivo bioimaging.

## Teaser

Terahertz imaging for noninvasive measurements.

## INTRODUCTION

Because of its unique properties, imaging and analysis with terahertz (THz) radiation have attracted much attention in recent years (*1*–*3*). For example, the transparency of most nonconductive materials in the THz range is extremely useful for systems inspection (*4*) and allows THz measurements to uncover the material composition and substructure of paintings, murals, or frescoes (*5*). The nonionizing photon energies are of interest to medical imaging applications, where resonant Debye relaxation of small molecules, such as water, gives rise to useful image contrast (*6*, *7*). However, unlike the visible domain, the THz regime is plagued by a lack of materials suitable for the construction of cheap and reliable focal plane imaging arrays, giving rise to the “THz gap” (*3*). Furthermore, because of the long wavelengths (0.15 to 1.5 mm), THz imaging is severely handicapped by the diffraction limit, restricting biological imaging, for example, to large structures such as organs (*8*, *9*). Therefore, there has been tremendous effort to develop subwavelength THz imaging techniques. These typically rely on some forms of raster scanning of a local modulator (*10*–*14*), or of the THz detector itself (*15*–*21*), in the near field. Undoubtedly, the most impressive THz imaging resolution has been achieved using tip scattering of near fields, where imaging of single nanoparticles is possible (*19*). Although these straightforward scanning approaches have yielded tremendous improvements in terms of resolution, they are also inherently slow, often invasive, and generally more suited to solid-state, conductive samples with well-defined interfaces (*18*, *19*).

In recent years, alternative imaging approaches have emerged, which use spatially controlled light, where the reflected, transmitted, or scattered radiation is recorded using a single-element detector (*22*–*26*). These approaches have both practical and economic advantages by completely dispensing with the need for slow mechanical scanning or expensive multipixel detectors. Further, these alternative imaging methods are compatible with compressed sensing (*27*), where one takes an *N* pixel image with *M* < *N* measurements, something unattainable by the imaging approaches of previous studies (*10*–*21*). Such single-element detection schemes have recently been demonstrated for far-field, diffraction-limited THz imaging with a typical resolution of ~1 mm (*28*–*34*), and it has been speculated that similar concepts could, in principle, be developed for efficient near-field THz imaging (*35*).

Here, we explicitly demonstrate near-field THz imaging using a single-element THz detector that can detect micrometer-sized fissures in a circuit board hidden on the underside of a silicon wafer. Our THz source is spatially modulated in the near field by a second optical source projected simultaneously onto a thin photoconductive modulator, which is itself placed in the near field of an object. Our technique combines many of the advantages of traditional THz imaging (such as transparency to nonconducting materials) with subwavelength spatial resolution. Because the spatial resolution of the imaging is fundamentally determined by the optical source, this approach retains the tantalizing prospect of noninvasive THz imaging with or without constraint of the diffraction limit.

## EXPERIMENT

**Fig. 1:**
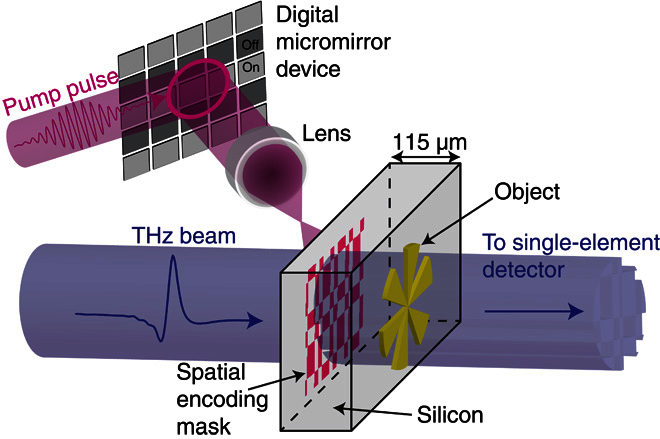
Schematic illustration of near-field, single-pixel THz imaging. The imaging scheme: An 800-nm pump pulse is spatially modulated and used to photoexcite a semiconducting wafer, which transfers the spatial encoding mask onto a coincident THz pulse. The subsequent THz pulse is then passed through an object onto a single-pixel detector.

**Fig. 2:**
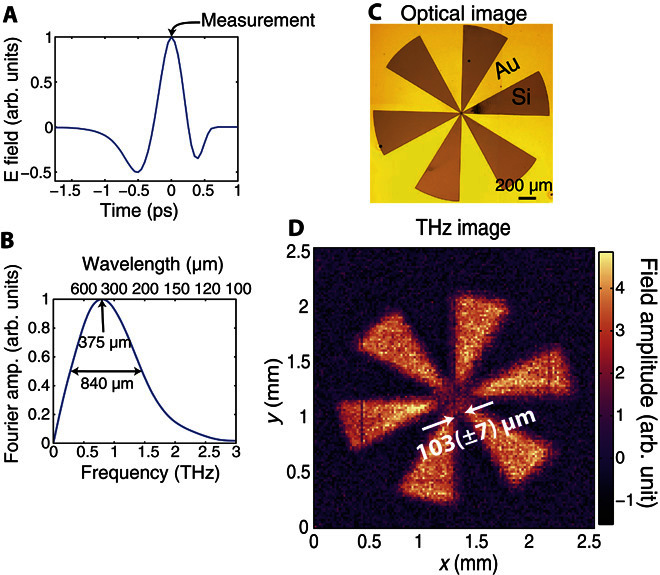
Pulsed THz imaging. (**A**) Electric field of our THz pulse recorded in the time domain using electro-optic sampling. The arrow shows the measurement point, at the peak of the THz field, for which images are recorded. (**B**) Normalized Fourier transform of our THz pulse. The central wavelength is approximately 375 μm, with a full width at half maximum of 840 μm. (**C**) Optical image of a resolution test target. Au marks the regions spanned by the gold film, whereas the regions that are marked “Si” show the exposed silicon wafer. (**D**) Image (128 × 128 THz) of the resolution test target in (C) obtained via a full set of Hadamard masks. The pixels are 20 μm in size. The arrows indicate the imaging resolution, evaluated as the maximal distance between the arms of the cartwheel for which the image contrast is diminished due to diffraction.

**Fig. 3:**
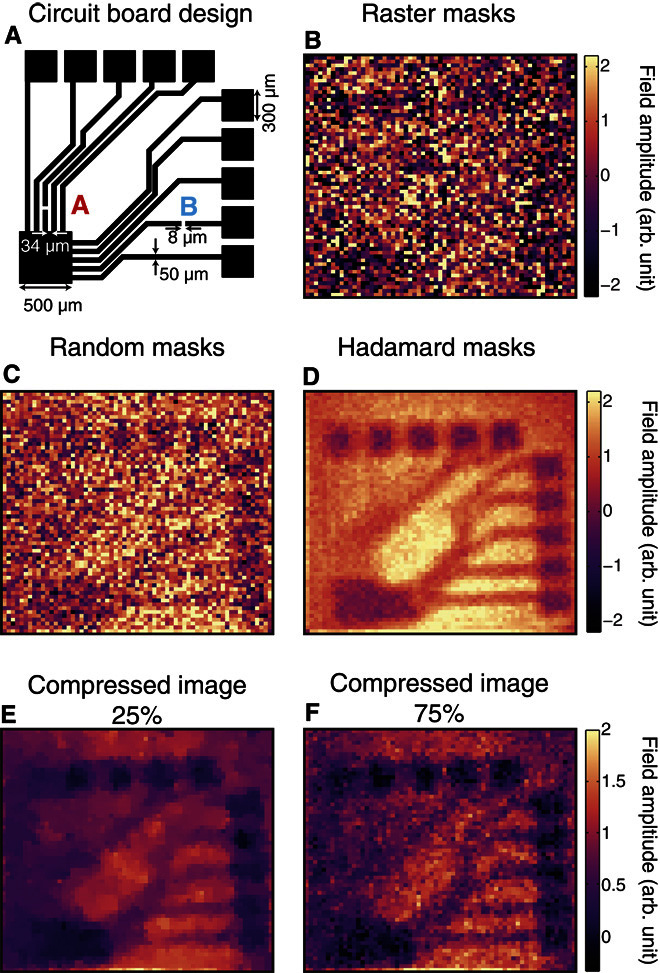
Hadamard versus random versus raster imaging. (**A**) Circuit board design, where black indicates conducting, metallic regions. The individual wires are 50 μm in width, and 8-μm breaks have been introduced at points marked by the letters A and B. (**B**) Image acquired using raster scanning of a single opaque pixel. (**C** and **D**) Comparison of the same image acquired with a full set of masks derived from random and Hadamard matrices, respectively. (**E** and **F**) Compressed images obtained via random masks, where the number of measurements is 25% (E) and 75% (F) of the total number of pixels (we use a total variation minimization image recovery algorithm; see section S6 for more details). In all images, the THz electric field is polarized horizontally, and the number of pixels is 64 × 64 with 40-μm pixels. The signal acquisition time for a single measurement is 500 ms. Because of the considerably larger noise in the measurement, we have scaled the image in (B) by 0.25 to use the same color scale as in (C) and (D).

Our imaging setup is illustrated in Fig. 1 (a more detailed schematic is shown in fig. S1). We use a time-domain measurement of a broadband THz pulse (0.2 to 2 THz; Fig. 2, A and B). To spatially modulate our THz beam, we shine a coincident 800-nm, 100-fs pump pulse onto a highly resistive silicon wafer (1000 ohms·cm, 115 μm thick). The pump pulse itself is structured into binary spatial intensity patterns by a standard micromirror device (*36*). When these patterns are projected onto the silicon wafer, the photoexcited regions are rendered conductive (see section S2) and thus also opaque to the coincident THz radiation (*37*). Moreover, because we record the THz transmission immediately following photoexcitation, before processes such as electron diffusion take place (see Materials and Methods), the spatial pattern encoded in the 800-nm pulse is directly transferred to the THz pulse without smearing or broadening of spatial features. The patterned THz pulse then propagates through ~115 μm of silicon before interacting with a sample positioned on the hidden side of the wafer, after which, we record the far-field transmission (see Materials and Methods). By spatially encoding a beam of THz radiation with binary intensity patterns, an image can be formed by analyzing the THz radiation transmitted or scattered by an object using a single-pixel detector (see Materials and Methods and section S3) (*26*, *38*). Further, because the distance traveled by the THz pulse before encountering the object is relatively small compared to the wavelength, we can record an image before far-field Fraunhofer diffraction occurs. In Fig. 2D, we show a measured THz image of the metallic cartwheel shown in Fig. 2C. A cartwheel is chosen as an object here because it contains ever increasing spatial frequencies toward the center of the wheel, allowing us to estimate the resolution of our image. The white arrows in Fig. 2D indicate an estimate of the imaging resolution, evaluated as the minimal distance between the arms of the cartwheel for which the image contrast is not diminished due to diffraction. From this, we find a resolution of 103 (± 7) μm, significantly smaller than the 375-μm peak wavelength of our THz pulse (see fig. 2B). Using scalar near-field diffraction theory (*39*), we can explicitly show that the resolution expected for our measurement is ~95 μm (see fig. S4), in agreement with our experimental estimate. Note that this resolution is by no means a fundamental limit and is determined primarily by the finite thickness of the photomodulator (the silicon wafer). The approach outlined above combines many of the advantages of traditional THz imaging, such as transparency to nonconducting materials (*40*), with subwavelength spatial resolution provided by the optical modulation. This makes it particularly suitable for imaging small structures buried beneath visibly opaque, nonconducting materials. We now demonstrate a potential application: imaging of a printed circuit board, hidden on the underside of a silicon wafer (see Fig. 3A for the design with dimensions).

## Single-element imaging approaches

We start by addressing the strategies by which we can record the data necessary to reform an image. The most straightforward way to obtain an image is to illuminate each pixel sequentially, as in raster scanning, and record the THz signal for each pixel. With this approach, the resulting image, shown in Fig. 3B, contains much noise arising from both our THz generation and detection. However, more complex multiaperture masking schemes can be devised using binary matrices for illumination. These multipixel illumination schemes offer the advantage of minimizing the effect of detector noise by using more light in each measurement (*26*). In terms of the choice of masks, we can use random binary masks, as used in compressed sensing (*27*). However, provided that one has a stable light source, the best results are usually given by masks derived from Hadamard matrices (*41*), namely, the masks form an orthonormal set that minimizes the mean squared error in each image pixel (*26*). In Fig. 3 (B to D), we compare images formed using raster, random, and Hadamard masks measured under identical conditions. For fair comparison, we compose our random masks from elements of 1s and −1s, akin to Hadamard masks. The raster images are averaged for twice as long to keep the measurement time equal. Although the multipixel schemes offer a clear advantage over sequential raster scanning, we also observe a clear superiority of Hadamard over random masks when we use a simple image reconstruction algorithm. To construct our images in Fig. 3 (B to D), we sum the masks with each one weighted by the detector readout for that mask. This algorithm is advantageous because of its fast computation (<100 ms), and for the Hadamard case, it recovers the exact solution. A more in-depth comparison for various image sizes is carried out in section S5.

The approach described above requires *N* measurements to obtain an *N* pixel image. However, more sophisticated image recovery algorithms have been developed by the field of compressed sensing (*27*), allowing one to image using *M* < *N* measurements. For this, we use a total variation minimization algorithm to recover our compressed images (see section S6 for details). In Fig. 3 (E and F), we show the effect of decreasing the number of measurements when sampling our circuit board with random masks. The structure of the circuit board can be observed even when only 25% of the measurements are used (Fig. 3E). However, some of the fine detail is missing because of undersampling. As the number of measurements are increased, the images begin to resemble the image measured using Hadamard masks in Fig. 3D. However, here, the image quality is primarily determined by the level of postprocessing that one performs, as discussed in section S6. Although compressive imaging can cut down measurement time, it does so at the cost of postprocessing and image detail. Moreover, with incomplete measurement sets, we lack the spatial resolution to see the fine image structure. In the remainder below, we therefore discuss the results obtained with Hadamard imaging.

## Application

**Fig. 4:**
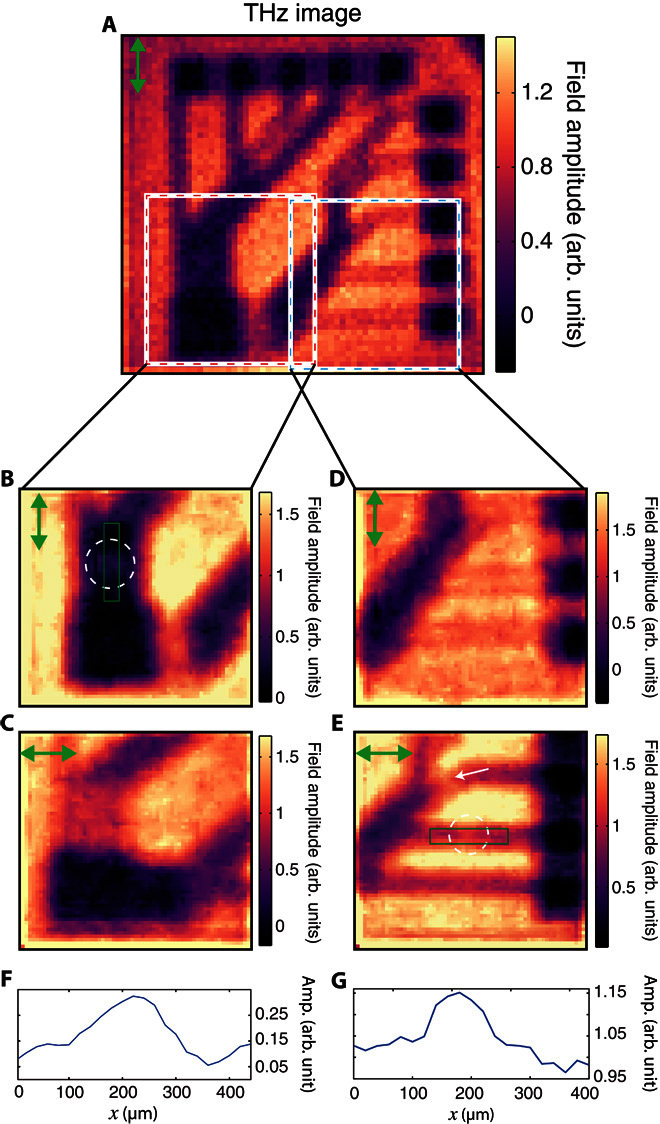
Imaged polarization effects. (**A**) Images (64 × 64) of circuit board in Fig. 3A with vertical polarization. Pixels are 40 μm. We see that the contrast of each of the individual wires in the circuit depends on the THz polarization, with the highest contrast seen for polarization parallel to the wires. (**B** to **E**) Images (64 × 64) of the square regions in (A). Polarization is shown by the green arrow on the top left corner of each picture. Pixel size is 20 μm, and images have been denoised using the algorithm described in section S7. We see that the very subwavelength wiring breaks [marked by circles in (B) and (E)] give rise to transmissive regions in the THz image when the THz polarization is parallel to the wire. In (E), the diagonally orientated wire (indicated by the white arrow) also shows low contrast. Every image has been obtained via a full set of Hadamard masks. (**F** and **G**) Line plots through the 8-μm gaps in (B) and (E) with amplitude and space on the vertical and horizontal axis, respectively. The spatial coordinates of the plots are indicated by green rectangles in (B) and (E).

Our THz source is linearly polarized; thus, we can expect effects due to the polarization boundary conditions. In particular, the electric field component parallel to the interface of a good conductor must approach zero. These effects are particularly prominent because of the subwavelength nature of the conducting features in our circuit board (the thickness of the conducting wires is 50 μm, and the separation between the individual wires at some locations is ~30 μm). In Fig. 4A, we show a THz image of the circuit board in Fig. 3A as measured with vertical THz polarization. We see that the subwavelength conducting wires are more clearly observed when the THz radiation is parallel to the wires. The biggest difference is seen in the conducting tracks that emerged from the large 500-μm square at the bottom left corner of Fig. 4 (B and C). Here, the small separation of the wires resembles a wire grid polarizer, with the transmission at its lowest (and image contrast at its highest) when the polarization is parallel to the wires.

Although such effects may be seen as a disadvantage, for example, by limiting the resolving capacity for some metallic features, we discuss below how we can use polarization sensitivity to our advantage by using it to detect very subwavelength features. To this end, we have introduced very small (≤8 μm) fissures in wires at two points marked by A and B in the circuit diagram in Fig. 3A. In Fig. 4 (B and E), these fissures appear as marked increases in the THz transmission amplitudes at the points identified by dashed circles. We also mark line plots through these gaps over the regions marked in green in Fig. 4 (F and G). Note that, to better distinguish these subwavelength features, Fig. 4 (B and C) has been denoised using the algorithm outlined by Edgar *et al*. (*42*) (see section S7 for a comparison of different filtering approaches). Note that the observed increase in THz transmission is considerably larger than one would expect solely from the reduced coverage of gold, and arises from a relaxation in the parallel field boundary condition because of the presence of the fissure. The subwavelength fissures are not visible when the polarization is perpendicular to the wires, as shown in Fig. 4 (C and D). Therefore, one can not only identify the orientation of the wiring using our approach but also detect extremely subwavelength defects in circuitry hidden beneath an optically opaque silicon.

## CONCLUSION

We have demonstrated noninvasive, subwavelength THz imaging using a single-pixel detector. Using subwavelength (~λ/4) resolution, we show a proof-of-principle application where we image a printed circuit board on the underside of a 115-μm-thick silicon wafer and show how polarization sensitivity can be used to detect subwavelength breaks in its thin conducting tracks. Although the resolution achieved here is limited by the thickness of the Si wafer, it is interesting to note that the fundamental limit for resolution in this approach, if one could use a thin film photomodulator, is the visible diffraction limit. Further, with potential for significantly greater acquisition rates, using faster (direct band gap) photomodulators, the imaging approach discussed here gives rise to several intriguing prospects, such as imaging of conducting channels in biological systems.

## MATERIALS AND METHODS

An amplified 800-nm (100-fs) Ti-sapphire femtosecond laser running at a repetition rate of 1050 Hz was used to power a THz time-domain spectrometer. The THz pulses were generated and detected, using optical rectification and electro-optic sampling, respectively (*43*, *44*), in ZnTe crystals. The femtosecond pulses also provided an optical modulation beam with fluences of 107 μJ/cm^2^. The pump pulse was spatially modulated via a digital micromirror device (DLP3000 with the DLP LightCrafter from Texas Instruments, with the original light-emitting diode illumination optics removed to allow direct access to the micromirrors) and a single lens (focal length, 75 mm) to project a binary pattern on the surface of a high-resistivity silicon wafer (1000 ohms·cm, 115 μm thick). The modulation pulse was coordinated with a collimated THz pulse in both space and time to arrive coincident pulse at the front interface of the silicon wafer. Note that the femtosecond laser spot was expanded to around four times the size of the micromirror array so that any effects of spatial variation in intensity are negligible. However, the THz spot was only slightly larger than the imaging field of view, which introduces some slow variation in intensity across our images. We achieved ~90% modulation depth via the plane photoexcitation of the Si wafer. We measured the THz transmission within a ~5-ps window after photoexcitation; hence, carrier diffusion can be neglected, as shown in section S2, and thus, our spatial pattern was directly imprinted onto our THz beam. The ultrafast synchronization between the 800-nm pump and the THz pulse was one of the key developments from the work of Zhao *et al*. (*21*), which has allowed us to access the subwavelength imaging regime. For our images, we recorded the peak of the THz pulse (Fig. 2A) transmitted through a sample, placed directly after the silicon modulator, in the far field. This gives a spectrally averaged weight to our measurements, centered around the peak spectral wavelength of 375 μm (see Fig. 2B). In principle, one can take images at all temporal points of the THz pulse and thus can obtain full spectral information. Note that we recorded modulation of the THz transmission, and not the transmission itself, because of the excitation of the photomodulator. To obtain an image, we recorded a total of *N* THz transmission measurements for *N* distinct spatial encoding masks. In matrix notation, this can be represented as Φ = *W*Ψ, where Φ is a vector of the sequential measurements made, *W* is a measurement matrix where the (*i*,*j*)th entry determines the value of the *j*th mask pixel in the *i*th measurement, and Ψ is an *N* pixel image of the object. The image can be obtained by inverse matrix multiplication: Ψ = *W*^−1^Φ (see section S3 for more information), but other methods exist if the matrix cannot be inverted (*27*). Our binary transmission masks had either opaque or transmissive pixels, meaning they were described by 1s and 0s. To preserve the orthonormality of Hadamard matrices, which were composed of +1s and −1s, we carried out sequential measurements of a mask directly followed by its inverse and recorded the difference in THz transmission for these measurements via a lock-in amplifier. This differential measurement is described by matrices with elements of +1 and −1 as outlined by Davis (*38*), and in fig. S3, we explicitly showed that this resulted in a favorable improvement in our images. The signal acquisition time for each mask was 50 ms. Note that for raster images, the THz transmission modulation measured in the experiment was for with and without mask (rather than for positive and inverse masks). Therefore, we averaged twice as long for raster imaging to achieve fair comparison. Note that the fundamental switching rate in our measurements was determined by the low repetition rate of the laser (1050 Hz). In principle, using a higher repetition rate laser could greatly increase the acquisition rate, which will be ultimately limited by the recovery time of the (MHz) photomodulator.

Samples were fabricated on the rear interface of the silicon wafer using 250-nm gold films deposited via thermal evaporation. A 5-nm layer of chrome acted as the adhesion agent between the silicon and the gold. Image patterns were wet-etched in the gold layer using electron beam lithography. At THz frequencies, the response of good conductors, such as gold, was essentially dispersionless. To investigate polarization effects in the experiment, samples were rotated, whereas the horizontal THz polarization remained fixed. All experiments were performed at room temperature.

## Supplementary Material

http://advances.sciencemag.org/cgi/content/full/2/6/e1600190/DC1

## SUPPLEMENTARY MATERIALS

Supplementary material for this article is available at http://advances.sciencemag.org/cgi/content/full/2/6/e1600190/DC1 section S1. Experimental schematics section S2. The silicon photomodulator section S3. Single-pixel detector imaging theory section S4. Scalar diffraction from two slits section S5. Signal with increasing number of pixels section S6. Total variation minimization reconstruction section S7. Image filtering fig. S1. Schematic of time-domain THz spectrometer. fig. S2. THz spectroscopy. fig. S3. [1, −1] versus [1, 0] masks. fig. S4. Diffraction from two slits. fig. S5. Increasing image size. fig. S6. Total variation minimized images. fig. S7. Unfiltered and filtered images. References (*45*–*55*)
