# Supplementary material for: Noninvasive, near-field terahertz imaging of hidden objects using a single-pixel detector
Source: Sci Adv. 2016 Jun 3;2(6):e1600190. doi: 10.1126/sciadv.1600190 (PMC4928995; doi:10.1126/sciadv.1600190)
Supplement: http://advances.sciencemag.org/cgi/content/full/2/6/e1600190/DC1 [file supp_2_6_e1600190__index.html]

Science Advances | Science Advances

## Supplementary Materials

**This PDF file includes:**

- section S1. Experimental schematics
- section S2. The silicon photomodulator
- section S3. Single-pixel detector imaging theory
- section S4. Scalar diffraction from two slits
- section S5. Signal with increasing number of pixels
- section S6. Total variation minimization reconstruction
- section S7. Image filtering
- fig. S1. Schematic of time-domain THz spectrometer.
- fig. S2. THz spectroscopy.
- fig. S3. 1, −1 versus 1, 0 masks.
- fig. S4. Diffraction from two slits.
- fig. S5. Increasing image size.
- fig. S6. Total variation minimized images.
- fig. S7. Unfiltered and filtered images.
- References (*45–55*)

Download PDF

**Files in this Data Supplement:**

- Adobe PDF - 1600190\_SM.pdf
