# Supplementary material for: Noninvasive, near-field terahertz imaging of hidden objects using a single-pixel detector
Source: Sci Adv. 2016 Jun 3;2(6):e1600190. doi: 10.1126/sciadv.1600190 (PMC4928995; doi:10.1126/sciadv.1600190)
Supplement: http://advances.sciencemag.org/cgi/content/full/2/6/e1600190/DC1 [file 1600190_SM.pdf]

Science Advances

AAAS

advances.sciencemag.org/cgi/content/full/2/6/e1600190/DC1

Supplementary Materials for

Noninvasive, near-field terahertz imaging of hidden objects using a single-pixel detector

Rayko Ivanov Stantchev, Baoqing Sun, Sam M. Hornett, Peter A. Hobson, Graham M. Gibson, Miles J. Padgett, Euan Hendry

Published 3 June 2016, *Sci. Adv.* **2**, e1600190 (2016)
DOI: 10.1126/sciadv.1600190

The PDF file includes:

- section S1. Experimental schematics
- section S2. The silicon photomodulator
- section S3. Single-pixel detector imaging theory
- section S4. Scalar diffraction from two slits
- section S5. Signal with increasing number of pixels
- section S6. Total variation minimization reconstruction
- section S7. Image filtering
- fig. S1. Schematic of time-domain THz spectrometer.
- fig. S2. THz spectroscopy.
- fig. S3. [1, −1] versus [1, 0] masks.
- fig. S4. Diffraction from two slits.
- fig. S5. Increasing image size.
- fig. S6. Total variation minimized images.
- fig. S7. Unfiltered and filtered images.
- References (*45*–*55*)

section S1. Experimental schematics

As stated in the main text, our THz pulses are generated and detected within a Terahertz time-domain spectrometer (THz-TDS) (*43*, *44*). The fundamental layout of the setup is shown in fig. S1. In its essence, a beam of femtosecond optical pulses is split into three beams: generation, detection and excitation. The first is used to generate a picosecond THz pulse, we use optical rectification in a ZnTe crystal (*45*), which then passes through the sample under investigation. Our THz beam is collimated and collected by 90° off-axis parabolic mirrors made from aluminum, both with a 2.5 cm focal length and 2.54 cm diameter. The second beam is used to detect the time profile of the THz waveform. This is achieved by temporally overlapping the much longer THz pulse with the very short detection pulse. The difference in pulse durations, allows one to discretely sample the terahertz temporal profile by varying the path lengths with an optical delay line (typical THz transient and detection pulse envelope shown in fig. S2A). The electric field amplitude is extracted via electro-optic sampling in a ZnTe crystal (*46*). The third beam is used to photoexcite the sample. Additionally, our excitation beam is spatially modulated via a digital micromirror device (DMD) and a lens so as to project any binary pattern onto our sample.

We use a single +7.5 cm focal length lens to project the binary patterns from the DMD onto our silicon, with a magnification of -0.66. Our DMD (DLP3000 used on a DLP Lightcrafter from Texas Instruments) has square mirrors of size 15.2 μm, hence we are limited to projecting squares of size ~10 μm. Using the Rayleigh lens formula, $\theta = 1.22\lambda/D$, our 800nm pulses have a diffraction limited resolution of ~5.4 μm at our imaging plane.

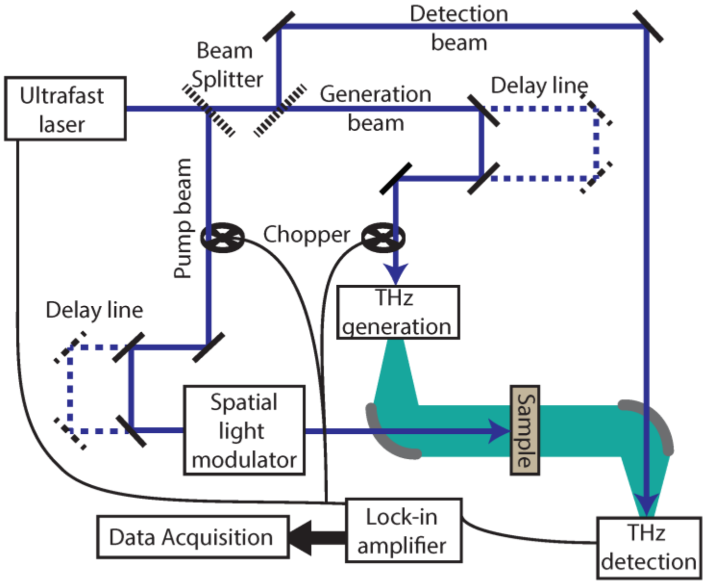

fig. S1. Schematic of time domain THz spectrometer. A beam of ultrashort optical pulses leave an ultrafast laser. The beam is split into three beams: generation, detection and excitation. A chopper is placed in the detection or generation beams, depending on the needs of the experiment. Parabolic mirrors are used to collect and collimate the THz radiation.

section S2. The silicon photomodulator

We photoexcitate electron-hole pairs in silicon using ultrafast (90 fs) pulses with a wavelength of 800nm. The photoexcited dielectric function of silicon can be described by the Drude model (*37*, *47*)

$$\varepsilon(\omega) = \varepsilon_b - \frac{\omega_p^2}{\omega^2 + i\omega / \tau_c} \quad (1)$$

where ε_b=11.7 is the background dielectric permittivity due to the bound electrons, τ_c is the average collision time with typical values $\tau_c \approx 0.5$ ps for the excitation energies used here (47), ω_p is the plasma frequency defined as $\omega_p^2 = n_c e^2/\varepsilon_0 m^*$ with e being the electron charge, ε_0 free space permittivity and $m^*=0.26m_e(0.37m_e)$ the effective mass of electrons (holes) (*48*).

The primary modulating parameter in the equation above is the density of carriers, n_c. Carriers are generated by pulses running at a repetition period of 1ms, considerably longer than the silicon carrier lifetimes of ~25 μs (*49*), thus we neglect sample heating. Moreover, since the THz pulse arrives ~5 ps after photoexcitation, as shown in fig. S2C, we can also neglect carrier recombination and diffusion. The key variable to determine is therefore the mean carrier-carrier distance. For the 5 ps in-between photoexcitation and the arrival of the THz pulse, we can calculate the mean square displacement of carriers, $\langle x^2 \rangle = 6Dt$, where D is the diffusion coefficient of our electron (hole) charge carriers. We use the Einstein–Smoluchowski relation, $D=\mu_q k_B T/q$, where μ_q is the mobility of charge carriers given by $\mu_q = q\tau_c/m^*$ (*50*), to obtain mean displacements of $\sqrt{\langle x^2 \rangle}$=506 nm(425 nm) for our photo-electrons (holes). Since the diffusion lengths are considerably smaller than the penetration depth of the photoexcitation light (~11 μm (*51*)), we can neglect carrier diffusion from our considerations. The carrier density is then given directly by the absorbed photon density. This gives $n_c = 2P\rho/d\hbar\omega_l$ where P is our pulse energy per unit area (104 μJ/cm^2), ρ=0.7 is the Fresnel transmittance of Si at our excitation wavelengths, $\hbar\omega_l$ is the photon energies of the pump light, d is the penetration depth ($d \approx 11$ μm (*51*) for our wavelengths) and the factor of 2 accounts for the electron-hole pair.

Entering these values, we obtain a plasma frequency of 81THz with ε(1THz) = -138+ 48i for our photoexcited silicon. In other words, we generate a THz material with a negative real and positive imaginary part to the dielectric function, the characteristics of a lossy conductor.

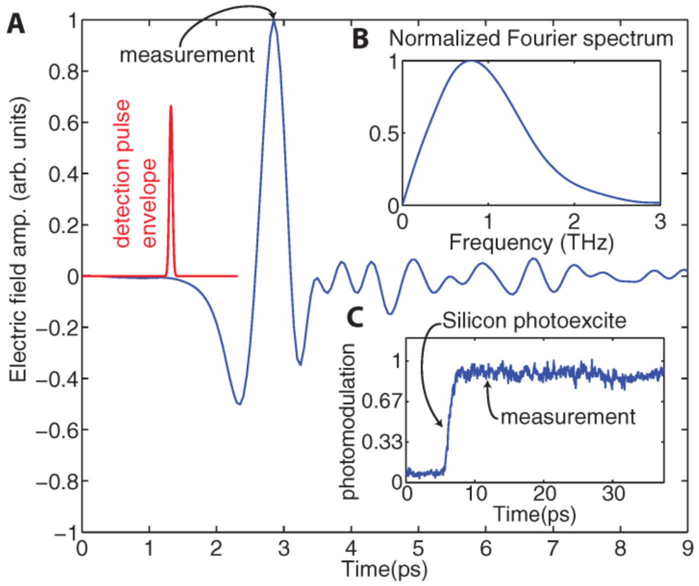

fig. S2. THz spectroscopy. (A) Blue: typical THz pulse detected by our system. Red: envelope of detection pulse used to discretely sample the THz waveform. Measurement arrow points to the THz amplitude we detect for each individual mask readout. Oscillations after main THz pulse are due to water vapor in the background environment. **(B)** Fourier spectrum of the THz pulse with water vapor oscillations zeroed out. **(C)** Modulated THz transmission due to the photoexcitation of a silicon wafer normalized to the THz transmission. Measurement arrow indicates where the measurements are made with respect to silicon photoexcitation (~5 ps after).

section S3. Single pixel detector imaging theory

The THz regime has the problem that detector arrays are difficult and expensive to manufacture (**3**), hence the need for imaging with single pixel detectors. The disadvantage to single pixel imaging is that it typically requires longer acquisition time compared to focal plane imaging arrays, due to the measurements being taken sequentially rather than in parallel. Here we are concerned with obtaining the spatial transmission function of some object using a single pixel THz detector. The simplest solution is to raster scan a single aperture to obtain the transmissivity pixel by pixel. However, if this aperture is made smaller and smaller, the detected signal is reduced and eventually one will run into detector noise.

One could sample more than one aperture simultaneously to increase the detected signal level in order to overcome detector noise, an idea which seems to first originate in 1935 with Yates (*52*). To do this, however, introduces extra calculation difficulties as the measured intensity is due to the sum of the scanning apertures. Therefore, the locations of the scanning apertures in each measurement must form a set of simultaneous equations which can be solved exactly for the individual pixels of the object's transmission function. The information regarding the location of the scanning apertures is held in mask configurations.

We now consider the construction of an N-pixel image Ψ. Our i^{th} measurement, ϕ_i, is the dot product of our object transmission function and the ith mask configuration, mathematically expressed as

$$\phi_i = \sum_{j=1}^{N} w_{ij}\psi_j \tag{2}$$

where w_{ij} holds the spatial information of the ith mask and ψ_j is the jth pixel of the image. As stated in the main text, this can be represented by the matrix equation $\Phi=W\Psi$, where the rows of W are reformatted into the projected masks. For invertible matrices W, the image vector Ψ can be obtained through matrix inversion $\Psi=W^{-1}\Phi$, which finally has to be reshaped into a 2D matrix of pixel values. Futher, the matrix equation $\Phi=W\Psi$ represents the image being expanded in some basis given by W. For this study we mainly use Hadamard matrices as our basis expansion, ie. W is a Hadamard matrix of order N (*26*). A Hadamard matrix H_n is defined as an $n\times n$ matrix of +1 s and -1 s with the property that the scalar product between any two distinct rows is 0 ie. each row is orthogonal to every other one. Thus H_n must satisfy

$$H_n H_n^T = H_n^T H_n = nI_n \tag{3}$$

where H_n^T is the transpose of H_n. This is a property that allows for easy image reconstruction as it is easy to see that $H_n^{-1} = H_n^T/n$. A more serious reason to construct masks from Hadamard matrices is that this basis minimizes the mean square error of each pixel in our image (*26*).

We have opaque masks that either block or transmit light ie. W contains only values of 1 s and 0 s and thus it is not a Hadamard matrix. However as outlined in (*38*), it is possible to obtain a fully orthogonal measurement matrix with such a system. Consider the H_2 matrix

$$H_2 = \begin{bmatrix} 1 & 1 \\ 1 & -1 \end{bmatrix} \tag{4}$$

The problem is that our measurement matrices can only have values of 1 and 0 corresponding to the mirrors being on or off respectively, but if we consider the following two matrices

$$G = \begin{bmatrix} 1 & 1 \\ 1 & 0 \end{bmatrix} \qquad V = \begin{bmatrix} 0 & 0 \\ 0 & 1 \end{bmatrix} \tag{5}$$

it is easy to see that $H_2=G-V$. Thus if we have two sets of measurement vectors each using one of the complementary sets of masks

$$\Phi_1 = G\Psi \qquad \Phi_2 = V\Psi \tag{6}$$

then subtraction of the second set gives the desired encoding matrix. This doubles the number of measurements required. However, if the complementary negative mask is projected immediately after its positive counterpart, one can eliminate an unwanted source of noise, namely low frequency source oscillations. In fig. S3, we compare the difference between using encoding masks derived from Hadamard matrices with [1, -1] and [1, 0] values. Here we can see that the image constructed from [1, 0] measurement has some artifacts created from low frequency THz source oscillations (indicated by the arrows), whereas the [1, -1] measurement have eliminated most of this type of artifact.

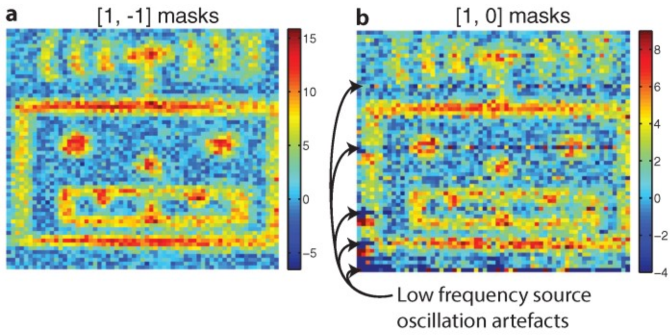

fig. S3. [1, –1] versus [1, 0] masks. Image obtained using Hadamard masks with values of [1, -1] in **(A)** and [1, 0] in **(B)**. Total number of measurements is 16384 for both pictures.

section S4. Scalar diffraction from two slits

As stated in the main text, the resolution of our imaging technique is limited by the thickness of our silicon photomodulator (115 µm). To calculate the diffraction in our system, we follow the method outlined in (*39*). Therein, Kowarz solves the 2D Helmholtz equation for positive z-space with all scatterers, sources and diffracting apertures being located in negative z-space. His electric field solution $U(x,z)$ is the sum of two parts, a homogeneous propagating contribution $U_h(x,z)$ and an evanescent component $U_i(x,z)$

$$U_h(x,z)=\int_{|u_x|\le 1} A(u_x)e^{iku_x x}e^{ikz\sqrt{1-u_x^2}}\mathrm{d}u_x \tag{7}$$

$$U_i(x,z)=\int_{|u_x|>1} A(u_x)e^{iku_x x}e^{-kz\sqrt{u_x^2-1}}\mathrm{d}u_x \tag{8}$$

where k is the free space wavenumber, ux is the directional wavevector in x and $A(u_x)$ is a spectral amplitude function that is the Fourier transform of scatterer's field distribution in the plane z=0, ie

$$A(u_x)=\frac{k}{2\pi}\int_{-\infty}^{\infty} U(x,0)e^{-iku_x x}\mathrm{d}x \tag{9}$$

This notation is known as the angular spectrum representation (*53*). We calculate the diffracted intensity distribution generated by two parallel slits with a field distribution given by

$$U(x,0)=K\left(\mathrm{rect}\left(\frac{x-a/2}{d}\right)+\mathrm{rect}\left(\frac{x+a/2}{d}\right)\right) \tag{10}$$

where rect is the rectangle function (*54*), d is the slits width, a is the slits' center to center separation and K is the incident wave amplitude. We are interested the intensity distribution, defined as $I(x,z)\equiv|U(x,z)|^2=|U_h(x,z)+U_i(x,z)|^2$, for various slit separations. To model our system more accurately, we sum all the frequency contributions of our pulses, with each frequency component weighted by our pulse spectrum (Fig. 2B**)** and a wavelength corresponding to our silicon dielectric.

In fig. S4 we plot the intensity distribution, on a parallel plane at a distance equal to our modulator thickness (115 μm), from two 20 μm slits for various slit separations. For separations <65 μm, the diffraction pattern is similar to that of a single slit. As the separation increases, the diffraction maxima arising from each slit become distinguishable. The white dashed line indicates the separation resolvable by the Rayleigh resolution criterion (*55*), corresponding to a resolution of ~95 μm which is well in agreement with our experimental estimate of 103(±7)μm.

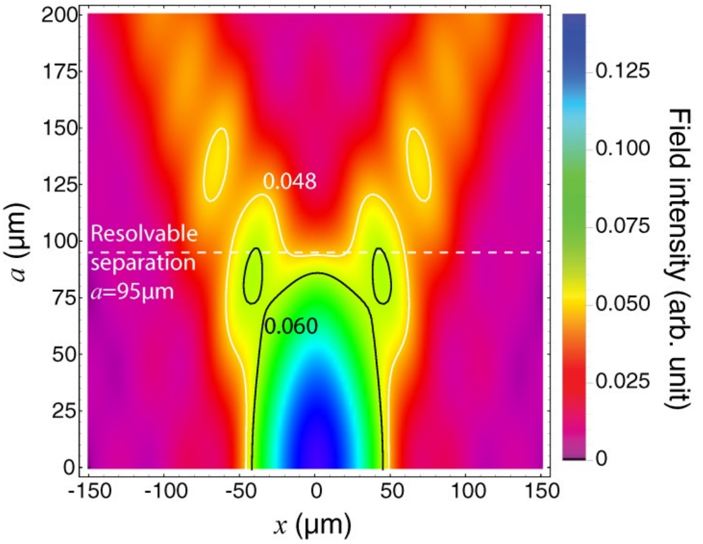

fig. S4. Diffraction from two slits. Intensity distribution (horizontal axis) from a slits as they separated apart (vertical axis). We plot the sum total intensity from an ensemble of spectrally weighted components that constitute our pulses (refer to experimental section for pulse spectrum). d=20 μm, z=115 μm in calculation.

section S5. Signal with increasing number of pixels

Here we investigate how experimental noise affects the three different masking schemes, outlined in the main text, as the number of pixels in the image is increased. For this, we take images under identical conditions (one after the other) of the circuit board in Fig. 3A with increasing number of pixels. Our results are shown in fig. S5. As shown in the main text, Hadamard masks have the most superior signal to noise followed by random masks and then by raster scanning. This is true for all image sizes. Raster scanning is most affected by detector noise due to the small signals emanating from a single aperture. On decreasing the aperture size, and increasing the number of pixels, image noise clearly increases. This effect is less significant for the multi-pixel approaches as these have larger associated signals. While multi-pixel patterns clearly have the benefit of increased signal to noise, the continual increase in the number of pixels leads to increased image noise, even for Hadamard imaging. This is because the signal from each individual pixel decreases as the number of pixels increase, and even though Hadamard matrices minimize the mean square error in each image pixel (*26*) they do not completely remove all noise. One should also note that we have noise in our THz source which further degrades image quality as the number of measurements required to form the image increases. Interestingly, random masks seem to fair best as the number of pixels increases. It can easily be shown that is an artefact caused by the simple reconstruction algorithm employed.

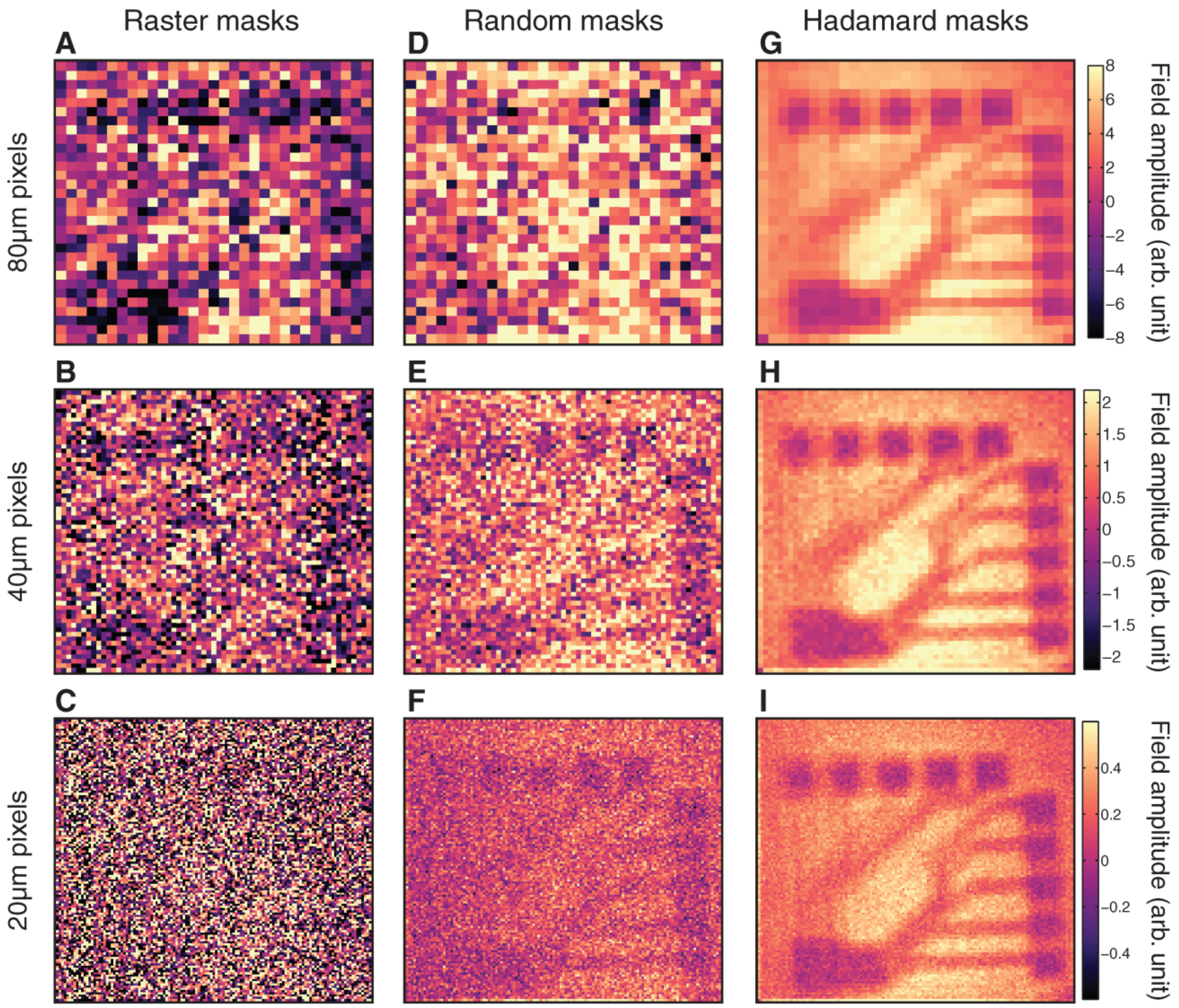

fig. S5. Increasing image size. (A to C) Images obtained using raster masks with increasing number of pixels from 32×32 to 64×64 and 128×128, respectively. **(D to I)** Images obtained using random (Hadamard) masks as number of pixels is increased from 32×32 to 64×64 and 128×128, respectively. The vertical lines seen in part **(C)** are associated with periodic changes in lab environment. Note **(A, B, and C)** have been scaled by 0.9, 0.25 & 0.1, respectively, so as to be plotted on the same scale as all other images.

section S6. Total variation minimization reconstruction

As stated in the main paper, we reconstruct our random mask images via a very simple algorithm where we sum the random masks with each one weighted by the detector readout for that mask. We use this simple algorithm due to its quick calculation times (~100 ms) and for the Hadamard case it recovers the exact solution. However, the idea of using masks constructed from random matrices originates from compressed sensing (*27*) where one usually minimizes the L1-norm of the vector (image in our case) that one wishes to sample in order to recover the correct solution. To obtain our images, we perform a total variation (TV) minimization. In other words, the mathematical problem is stated as

$$\begin{aligned} &\text{minimize} \quad \mathrm{TV}(\Psi) \\ &\text{subject to} \quad \|W\Psi - \Phi\|_2 \leq \gamma \end{aligned} \tag{11}$$

where W is our random measurement matrix, Φ is our vector of measurements, Ψ is the image we are interested in, γ is a variation relaxation parameter allowing us to determine how smooth the final image is and TV is the total variation of a 2D image defined as

$$\mathrm{TV}(x) \equiv \sum_i \sqrt{(\mathrm{D_h}x)_i^2 + (\mathrm{D_v}x)_i^2} \tag{12}$$

where x is a 2D image and $\mathrm{D}_{h,v}$ are the discretized gradient operators along the horizontal and vertical directions respectively. Our calculations were performed in Matlab 2013b using the L1-magic package.

This algorithm is more complicated (taking us ~100 s), however with it we obtain an image that has a significantly better signal to noise ratio as shown in fig. S6. Here, we see that Hadamard is still superior. This is partly due to the fact that Hadamard matrices minimize the mean square error in each image pixel (*26*) and partly due to the value of our relaxation parameter. In other words, we could further improve the quality of our random mask image with more careful considerations of our value for γ.

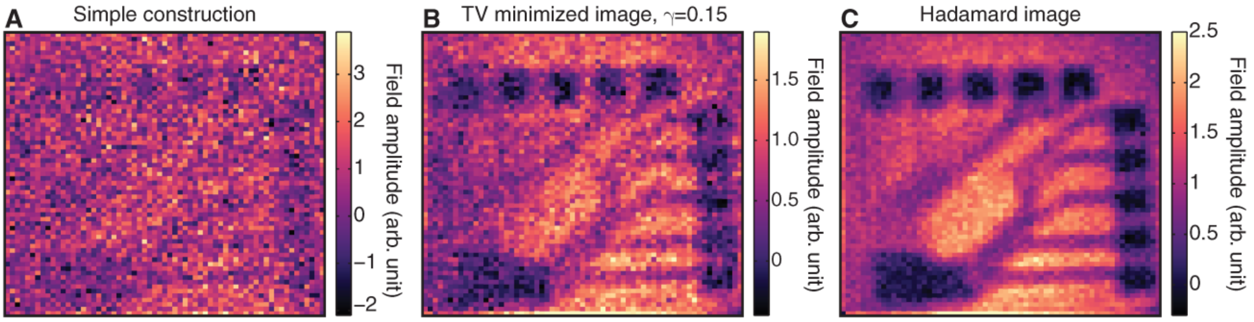

fig. S6. Total variation minimized images. 64×64 images of a circuit board where **(A)** and **(B)** have been obtained using random masks except **(A)** was constructed using our simple algorithm and **(B)** has been constructed via a total variation minimization algorithm from the same data. **(C)** Image of the same circuit board obtained via Hadamard masks.

section S7. Image filtering

In fig. S7A-D we show THz images of certain sections of the manufactured circuit board (see Fig. 3A for design). With these images we show that it is possible to observe, using the strong polarization effects shown in Fig. 4, highly subwavelength (8 μm) breaks along the conducting wires. Upon close investigation of fig. S7, one can see the breaks manifesting as small localized increases in field amplitude. However, due to the noise embedded in the measurement this is not immediately obvious.

The experimental errors were minimized and a total of ~2650 pulses were utilized for each measurement, which results in rather noisy images (fig. S7A-D). Hence we are left to reduce the noise in our images via post-processing. For this, we employ a spatial Fourier filter and a spatial

curvature denoising algorithm (outlined below). For Fourier filtering we use a Gaussian lowpass filter of size 15 with a standard deviation of 1.1, as implemented by the 'fspecial' command in MATLAB. These parameters were subjectively chosen based on subjective image quality. In contrast, the denoising algorithm, as outlined below and in (*42*), has no subjective input.

Our noise is embedded within the spatial-curvature of our images. If we minimize the spatial-curvature, then the noise will also be minimized. However, we do not want to remove image features not due to noise, thus we have to put a constraint to limit the minimization. The constraint should also be chosen to reflect the nature of the noise to be filtered; Gaussian noise in our case. For this reason, we look at the square of the difference between our denoised and original images. The denoised image is obtained by minimization of its cost function, C, given by

$$C = \frac{1}{N}\sum_{i=1}^{N}\left(\frac{\sum_{j=1}^{N}\left(w_{ij}\psi_j\right)-\phi_i}{\sigma_s}\right)^2 + \lambda\left(\left|\frac{d^2\Psi'}{dx^2}\right| + \left|\frac{d^2\Psi'}{dy^2}\right|\right) \tag{13}$$

where Ψ' is the image vector expressed in 2D format, σ_s is the standard deviation of the noise in the measurement of ϕ_i. The first term in eq. (13) represents a χ^2/N distribution of the image with respect to the measured data, and the second term represents the total spatial-curvature of the image. λ is the regularization parameter dictating the level of smoothing: larger values lead to smoother denoised images. The algorithm starts with a value of λ=1 and automatically increases this value to ensure that, once optimized, $\chi^2/N \approx 1$.

In fig. S7E-L we show the raw images in fig. S7A-D filtered by the two methods outlined above. Both methods considerably improve the looks of the images, and more importantly they preserve the features we wish to show: the breaks manifesting as small localized increases in amplitude.

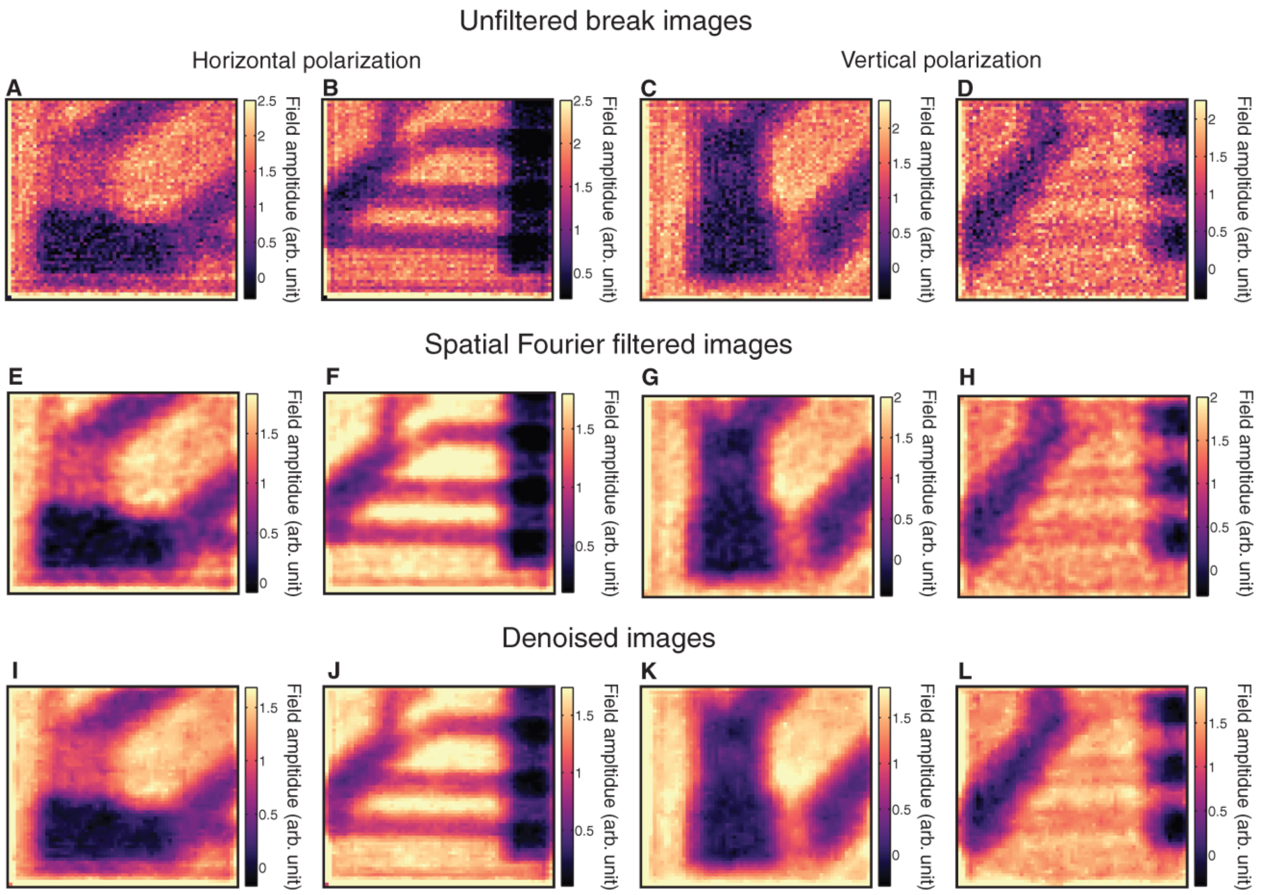

fig. S7. Unfiltered and filtered images. (A and **B)** Image of Break A and B, respectively, with horizontal polarization. **(C** and **D)** Image of Break A and B, respectively, with vertical polarization. **(E** to **L)** Fourier filtered (denoised) images of Breaks A and B from parts **(A** to **D)** respectively. Number of pixels is 64×64 with each pixel being 20 μm in size for all images.
